# Increased complications rates and inferior patient reported outcomes following total knee arthroplasty due to post-traumatic osteoarthritis with previous fracture treatment: a systematic review

**DOI:** 10.1007/s00167-023-07407-x

**Published:** 2023-04-25

**Authors:** Ioannis Syrikas, Cecilia Engbäck, Georgios Tsikandylakis, Ioannis Karikis, Neel Desai

**Affiliations:** 1 Department of Orthopaedics, NU-Hospital Group, Trollhättan/Uddevalla, Sweden; 2 Department of Orthopaedics, Institute of Clinical Sciences, Sahlgrenska Academy, University of Gothenburg, Gothenburg, Sweden; 3 Department of Orthopaedics, Sahlgrenska University Hospital, Mölndal, Sweden; 4 Department of Research and Development, NU-Hospital Group, Trollhättan, Sweden

**Keywords:** Posttraumatic osteoarthritis, Osteoarthritis, Total knee arthroplasty, Fracture, Patient-reported outcome measures, Complications

## Abstract

**Purpose:**

This study aims to present the existing literature relating to patient-reported outcome measures (PROMs) and complications in patients undergoing total knee arthroplasty (TKA) due to posttraumatic osteoarthritis (PTOA) with prior fracture treatment around the knee compared with patients who underwent TKA because of primary osteoarthritis (OA).

**Methods:**

A systematic review was undertaken and synthesised in accordance with the PRISMA guidelines by searching existing literature in the following databases: PubMed, Scopus, Cochrane Library and EMBASE. A search string according to the PECO was used. After analysing 2781 studies, 18 studies (5729 PTOA patients/149,843 OA patients) were included for a final review. An analysis revealed that 12 (67%) were retrospective cohort studies, four (22%) were register studies and the remaining two (11%) were prospective cohort studies. The mean Critical Appraisal Skills Programme (CASP) score was 23.6 out of 28, signifying studies of moderate quality.

**Results:**

The most frequently reported outcome measure were postoperative complications, reported in all eighteen studies. Intraoperative complications were reported in ten (4165 PTOA/124.511 OA) and patient-reported outcome measures (PROMs) in six studies (210 PTOA/2768 OA). A total of nine different PROMs were evaluated. As far as PROMs were concerned, the scores were inferior for PTOA but did not differ statistically from OA, except for one study, which favoured the OA group. Across all studies, postoperative complications were higher in the PTOA group, reporting infections as the most common complication. Furthermore, a higher revision rate was reported in the PTOA group.

**Conclusion:**

PROM analysis suggests that both patient groups benefit from a TKA in terms of functional outcome and pain relief, however, patient-reported outcomes could be inferior for PTOA patients. There is consistent evidence for increased complication rates following PTOA TKA. Patients undergoing TKA due to PTOA after fracture treatment should be informed about the risk for inferior results and refrain from comparing their knee function to patients with TKA after OA. Surgeons should be aware of the challenges that PTOA TKA poses.

**Level of evidence:**

Level III.

**Supplementary Information:**

The online version contains supplementary material available at 10.1007/s00167-023-07407-x.

## Introduction

Total knee arthroplasty (TKA) is widely regarded as an effective and safe surgical treatment in the management of osteoarthritis (OA), often offering pain-relief and functional improvement [41, 46]. The number of TKAs performed over the past few years has risen substantially, leading to this operation becoming one of the most frequently performed joint replacements worldwide [3, 22].

Knee injuries are a well-established major risk factor for the development of knee OA, along with obesity [12, 44], heredity factors [18] and ageing [50]. Studies report that 20–50% of patients with prior trauma develop OA, which is estimated to represent 12% of the total prevalence of symptomatic knee OA [7, 45]. This condition is also termed posttraumatic osteoarthritis (PTOA) and its aetiology is multifactorial; periarticular fractures, concomitant cartilage and meniscal lesions, changes in knee kinematics and homeostasis after anterior cruciate ligament (ACL) injury, to name but a few [4].

Although TKA is often an effective surgical treatment for primary OA, performing this procedure in patients with PTOA secondary to fracture injuries in and around the knee joint, treated with or without osteosynthesis, is more technically demanding [48]. There has been a growing interest about whether TKA in patients with previous knee fracture has inferior results compared with the results of TKA in the general knee osteoarthritic population [34, 37, 47].

Previous reviews have attempted to the report on outcome TKA due to PTOA. However, most of them have not explicitly compared PTOA with primary OA [5, 38]. Others included studies that did not specify the kind of knee injuries included in the PTOA group [23] or included only tibial plateau fractures [33, 42]. Little known about the TKA outcome due to PTOA after other types of fracture around the knee. The current systematic review was therefore designed to include a larger number of higher quality and more recent studies and aimed to compare the outcome of TKA in patients with PTOA, after treatment of any fracture around the knee, with the outcome of TKA after primary OA in terms of patient-reported outcome measures (PROMs) and complication rates. We hypothesised that PTOA due to previous fracture treatment leads to inferior PROMs and increased complication rates after TKA compared with patients undergoing TKA after primary OA.

## Methods

### Search strategy

An extensive search was performed in January 2022 in the following databases: PubMed, the Cochrane Library, Scopus and EMBASE with the assistance of librarians at the biomedical library of our institution. Studies published from January 2000 to January 2022 were included in the first search, while an updated search was performed in the same four databases in November 2022, using the same search syntax, to include more recent studies published until November 2022. The search syntax is presented in Supplementary file Appendix A. This study was reported and synthesised in accordance with the Preferred Reporting Items for Systematic Reviews and Meta-Analyses (PRISMA) guidelines from 2020 [32].

### Eligibility criteria

Articles in this systematic review had to match the following inclusion criteria described in the PECO (**P**atients-**E**xposure-**C**omparator-**O**utcome) framework. The population were adult patients undergoing TKA. The exposure was TKA due to PTOA after fracture management and the comparator was TKA due to primary OA. The outcomes were PROMs, as well as intra- and postoperative complications. Study designs could be randomised controlled trials, prospective comparative, register studies and/or retrospective comparative studies. Studies lacking comparators, studies on computer assisted TKA, unicompartmental knee arthroplasty, osteotomies around the knee, case reports, case series, book chapters and expert opinions, were excluded. The search covered human-based studies published in English between January 2000 and November 2022. Non-English texts, cadaver or animal studies were excluded.

### Study selection and data extraction

**Fig. 1:**
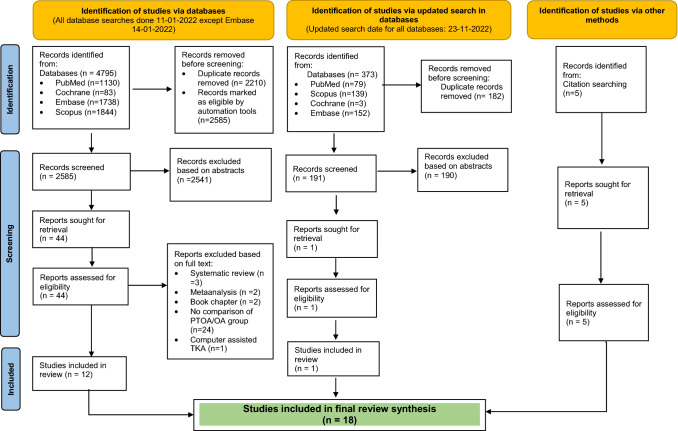
PRISMA flowchart illustrating inclusion and exclusion of studies for review

After completing the search, the articles were blindly and independently screened by two of the authors based on title and abstract in the initial stage and subsequently reviewed by full text to analyse whether or not they met the inclusion criteria. Disagreements regarding inclusion/exclusion were adjudicated and resolved through discussion with a third author. An automation tool called ¨Rayyan¨ [1] was used to expedite the initial screening of abstracts and titles. It is a free web tool using a process of semi-automation, while incorporating a high level of usability. Reference lists of applicable reviews [5, 23, 33, 38, 42] were reviewed to identify additional studies for the robust inclusion of pertinent literature, referred to as ¨citation searching¨ in the PRISMA diagram (Fig. 1). Once the articles were selected, data from each study were extracted independently by two authors. For each primary study, data were extracted and summarised in structured tables or plots using Microsoft Excel. The data items that were obtained from the included articles are presented in Table 1.

**Table 1:** Data items extracted from included papers

Study—related items	Patient-reported outcome measures	Intraoperative data & complications	Postoperative complications
Author	WOMAC	Tourniquet time	***Infections***
Year of publication	SF-12	Operative time	Deep infection & PJI
Title	KSS	Anaesthesia time	Superficial infection
Journal	KOOS	Blood transfusion	Irrigation and debridement
Volume	LEAS	Calculated blood loss	Sepsis
Issue	VAS	Ligament injury	Wound complications
Pages	IKS		***Soft tissue complications***
ISSN	OKS		Stiffness/athrofibrosis
DOI	Level of pain		MUA
Abstract			Patella instability
Study type			Peroneal nerve palsy
Period of patient recruitment			***Revisions***
Number of cases with PTOA/OA			TKA revision rate for all causes
Number of fracture cases in the PTOA group			TKA revision rate due to specific causes
Mean age at insertion of TKA			***Other complications***
Mean time from fracture treatment to TKA			DVT/PE
Mean follow-up time from TKA			Readmission rate 90 days postoperatively
Length of stay in the hospital after TKA			Periprosthetic fracture
			Mortality
			Periprosthetic fracture
			Additional procedure

*ISSN* International Standard Serial Number, *DOI* Digital object identifier, *PTOA/OA* The group of patients who underwent total knee arthroplasty due to posttraumatic osteoarthritis because of fracture around the knee joint/The group of patients who underwent total knee arthroplasty due to primary osteoarthritis without previous injury around the knee joint, *PJI* Periprosthetic joint infection, *PTOA* Posttraumatic OA, *TKA* Total knee arthroplasty, *MUA* Manipulation under anaesthesia, *ROM* Range of motion, *DVT* Deep venous thromboembolism, *PE* Pulmonary embolus, *KSS* Knee Society Score, *WOMAC* Western Ontario and McMaster Universities Osteoarthritis index, *SF-12* The Short Form (12) Health Survey, *VAS* Visual Analogue scale, *LEAS* Lower Extremity Activity Score, *KOOS* Knee Injury and Osteoarthritis Outcome Score, *OKS* Oxford Knee Score, *IKS* International Knee Score

### Methodological quality assessment of included articles

The methodological quality of the included studies was assessed using a modified version of the CASP checklist designed for cohort studies [2]. The CASP tool appraises three broad issues of each article involving fourteen items with total score of 28p and is regarded as an acceptable framework for quality assessment [26]. To interpret the total score (= 28p), a percentage was obtained based on the use of the checklist in previous articles [31]. A total score of 28p corresponds to high methodological quality, 27–22p moderate and ≤ 21p low methodological quality.

Using the CASP checklist for cohort studies, the majority of the included articles (*n* = 12) were rated as being of moderate quality [6, 13, 16, 17, 20, 21, 25, 29, 36, 40, 43, 49], two were rated as high quality [11, 19] and four were rated as low quality [10, 24, 27, 35]. The mean CASP score was 23.6p (range 16–28), indicating moderate quality studies. No eligible study was excluded due to a low CASP score. Instead, this tool was used to assess the extent to which each article contributed to the study aims. The lowest level of evidence in the included studies was III (Table 2).

**Table 2:** Assessment of the methodological quality of included studies

Study	CASP criterion^a^																Level of evidence ^c^
	1	2	3	4	5a	5b	6a	6b	7	8	9	10	11	12	Total score^b^	Methological quality	
Bergen et al. (2019)	2	2	2	2	1	2	2	2	2	2	2	2	2	2	27	**Moderate**	**3**
Dexel et al. (2016)	2	2	2	2	0	0	0	0	2	2	2	2	2	2	20	**Low**	**3**
El- Galaly et al. (2017)	2	2	2	2	2	2	2	2	2	2	2	2	2	2	28	**High**	**3**
Frisch et al. (2020)	2	2	2	2	0	0	2	2	2	2	2	2	2	2	24	**Moderate**	**3**
Houdek et al. (2015)	2	2	2	2	0	1	2	2	2	2	2	2	2	2	25	**Moderate**	**3**
Houdek et al. (2016)	2	2	2	2	0	0	2	2	2	2	2	2	2	2	24	**Moderate**	**3**
Kester et al. (2016)	2	2	2	2	2	2	2	2	2	2	2	2	2	2	28	**High**	**3**
Khoshbin et al. (2019)	2	2	2	2	2	2	2	2	1	1	2	2	0	2	24	**Moderate**	**3**
Kreitz et al. (2016)	2	2	1	2	2	1	2	2	2	2	2	2	2	2	26	**Moderate**	**3**
Lizaur-Utrilla et al. (2015)	2	2	2	2	0	0	2	0	2	1	2	2	2	2	21	**Low**	**2**
Lunebourg et al. (2015)	2	2	2	2	1	0	2	2	2	2	2	2	2	2	25	**Moderate**	**3**
Manrique et al. (2018)	2	2	2	2	0	0	2	0	0	0	0	2	2	2	16	**Low**	**3**
Miozzari et al. (2021)	2	2	2	2	0	0	2	2	2	1	2	2	2	2	23	**Moderate**	**2**
Phruetthiphat et al. (2021)	2	2	1	2	0	0	1	1	2	1	2	1	1	2	18	**Low**	**3**
Piedade et al. (2013)	2	2	1	2	1	1	2	2	2	2	2	2	2	2	25	**Moderate**	**3**
Scott et al. (2015)	2	2	1	2	0	0	2	2	2	2	2	2	2	2	23	**Moderate**	**2**
Stake et al. (2021)	2	2	2	2	1	1	2	2	2	2	2	2	1	2	25	**Moderate**	**3**
Wood et al. (2022)	2	1	2	2	2	1	1	2	2	2	2	0	2	1	22	**Moderate**	**3**

*CASP* Critical Appraisal Skills Programme

^a^1. Did the study address a clearly focused issue? 2. Was the cohort recruited in an acceptable way? Was the exposure accurately measured to minimize bias? Was the outcome accurately measured to minimize bias? 5(a) Have the authors identified all important confounding factors? 5(b) Have they take account of the confounding factors in the design and/or analysis? 6(a) Was the follow up of the subjects complete enough? 6(b) Was the follow up of subjects long enough? 7. What are the results of this study? 8. How precise are the results? 9. Do you believe in the results? 10. Can the results be applied to the local population? 11. Do the results of this study fit with other available evidence? 12. What are the implications of this study for practice?

^b^(I) Criterion completely met = 2; (II) Criterion partially met= 1; (III) Criterion not applicable/not met/not mentioned = 0. Total score 28 = high quality (H); 27–22 moderate quality (M); ≤ 21 low quality (L)

^c^The included studies do not clearly state the Level of Evidence (LoE), therefore, the LoE provided in this table was judged by the authors of the present review according to the described methodology of each study. Level 1: Randomised controlled trial; Level 2: Prospective cohort study; Level 3: Retrospective cohort study; Level 4: Case series; Level 5: Mechanism-based reasoning

### Methodology used to limit the risk of bias

To address the risk of bias, the population, the exposure, the comparators and the study’s outcome were clearly specified according to the PECO framework, defining the adopted research strategy of the review. To ensure that the assessments were thorough, consistent, and as objective as possible, we used a structured risk of bias assessment tool, the CASP cohort checklist. Finally, the PRISMA guidelines provided a methodical and structured framework, on which our review was based.

## Results

The first literature search (January 2022) yielded 4795 results. After screening for duplicates, 2585 articles remained. Following the application of eligibility criteria, 44 reports were sought for retrieval. A total of 12 studies were included in the review in the first phase of database search. Five more studies were identified through citation searching [13, 20, 21, 25, 35]. The updated search, yielded 191 additional studies, of which one [49] met the inclusion criteria, resulting in a total of 18 studies for the final synthesis of this review (Fig. 1).

### Study characteristics

The included studies comprised twelve retrospective, four register-based and two prospective cohort studies (Table 3). The studies reported on a total of 155,572 patients (range 87–68,349); 5729 (3.7%) in the PTOA group with prior fracture treatment and 149,843 (96, 3%) in the primary OA group. Two studies [16, 17] used the same cohort in the primary OA group and so this patient cohort was only counted once. In the PTOA group, 4001 (69.8%) patients had had their previous fracture around the knee treated with osteosynthesis, 265 (4.6%) patients had been conservatively and in 1463 (25.5%) patients, the method of their fracture treatment was not specified. Postoperative complications were the most frequently reported outcome measure, reported in all studies, followed by intraoperative complications, reported in ten studies [6, 10, 13, 19, 21, 35, 36, 40, 49], and PROMs reported in six studies [20, 24, 25, 35, 36, 40].

**Table 3:** Study characteristics of included studies

No	Study publication year)	Period of patient recruitment	Type of study	Number of cases in PTOA^a^/OA	Number of fracture cases in the PTOA^a^ group (%)	Mean age at insertion of TKA (PTOA^b^/OA)	Mean time from fracture treatment to TKA (years)	Mean follow-up time from TKA (PTOA/OA)	Length of stay in the hospital (PTOA^b^/OA) (days)	Outcome measures
**1**	Bergen et al (2019)	2005–2017	R	109/109	56 (60.5%)^d^	58.7/58.9 n.s.	N/A	3 m	2.85/3.03 n.s.	IC, PC
**2**	Dexel et al (2016)	2005–2013	R	170/1671	68 (40%)^d^ 19 (11.1%)^e^	65/71	24.1	N/A	8.2/8.5 n.s.	IC, PC
**3**	El- Galaly et al (2017)	1997–2013	REG	1421/51097	1421 (100%)	Age group<50 year, *n*=227/1377: 42/46 (***p*<0.001**)	N/A	Age group<50 year: 6.1 year/4.6 year	N/A	IC, PC
						Age group 50–70 year, *n*=771/24259: 60/62 (***p*<0.001**)		Age group 50–70 year:6 year/5.2 year		
						Age group>70 year, *n*=423/25461: 77/77		Age group>70 year: 5.8 year/5.1 year		
**4**	Frisch et al (2020)	2011–2017	R	130/130	26 (20%)^d^	60.7/59.6 n.s.	N/A	3 m	2.8/2.8 (***p* 0.019**)^c^	IC, PC
**5**	Houdek et al (2015)	1990–2012	R	113/19641	41 (36%)^d^ 14 (12%)^f^ 10 (9%)^g^ 48 (43%)^e^	67/(N/A)	N/A	6 year	N/A	PC
**6**	Houdek et al (2016)	1990–2012	R	531/19641	328 (62%)^d^ 203 (38%)^e^	62/(N/A)	4	6 year	N/A	PC
**7**	Kester et al (2016)	2010–2013	REG	674/67675	674 (100%)^d^	59.3/66.9 (***p* 0.001**)	N/A	3 m	In PTOA longer 0.56 days (***p* <0.001**)	IC, PC
**8**	Khoshbin et al (2019)	2007–2012	R	75/375	75 (100%)^d^	67.7/67.8 n.s.	19.3	93/88.2 m n.s.	N/A	PROM, PC
**9**	Kreitz et al (2016)	2008 - 2015	R	63/189	25 (39.7%)^d^	58.7/59.5	N/A	N/A	2.48/2.43 n.s.	IC, PC
**10**	Lizaur-Utrilla et al (2015)	2003–2009	P	29/58	22 (76%)^d^ 7 (24%)^e^	57.3/59.2 n.s.	6.9	6.4/7.1 year	N/A	PROM, PC
**11**	Luneborg et al (2015)	1998–2005	R	33/407	28 (84.8%)^d^ 5 (15.2%)^e^	69/72 n.s.	14	11 year	N/A	PROM, PC
**12**	Manrique et al (2018)	2007–2012	R	55/110	24 (43.6%)^d^	56.7/57.6	N/A	43.45/40.52 m	N/A	PC
**13**	Miozzari et al (2021)	2000–2016	REG	844/3101	75 (9%)^d^	67.3/72.5 (***p*< 0.001**)	N/A	8.6 year	N/A	PC
**14**	Phruetthiphat et al (2021)	2006–2012	R	136/716	32 (24%)	56.5/63.8 (***p*< 0.0001**)	N/A	8 year	3.1/2.9 (*p* 0.63)	PROMS, IC, PC
**15**	Piedade et al (2013)	1988–2005	R	231/1119	10 (4%)	71/72 (***p*< 0.01**)	N/A	45.6/43.2 m n.s.	N/A	PROMS, IC, PC
**16**	Scott et al (2015)	1997–2011	P	31/ 93	24 (77%)^d^ 7 (23%)^e^	65.7/66.3 n.s.	2	85 m	N/A	PROMS, IC, PC
**17**	Stake et al (2021)	2010–2019	R	823/1640	823 (100%)^d^	<50 year:97/191 50–60 year: 229/456 60–70 year: 290/579 70+ year: 207/414 n.s.	N/A	2year	N/A	PC
**18**	Wood et al (2022)	2010–2019	REG	1712/1712	1712(100%)^d^	63.5/63.6	N/A	2year	N/A	IC, PC

*N/A* Not applicable, *n.s.* Non-significant, *PC* Postoperative complications, *IC* Intraoperative data/complications, *PROM* Patient-reported outcome measures, *R* Retrospective, *P* Prospective, *REG* Register study, *y* Years, *m* Months, *n* Number of patients PTOA/OA, *bold numbers* p<0.05

^a^This group of patients may include not only previous fracture injuries around the knee but also other injuries such as ligament injuries, high tibial or distal femoral osteotomy, previous open arthrotomy or meniscectomy which could lead to posttraumatic osteoarthritis

^b^The group of patients with posttraumatic osteoarthritis exclusively due to fracture injury around the knee joint

^c^p value between PTOA^a^ and OA group

^d^Operative fracture management (osteosynthesis)

^e^Non operative fracture management

^f^Treated operatively with partial patellectomy

^g^Treated operatively with total patellectomy

The majority of included studies does not refer to the specific type of TKA implant used, however, in the studies that this information is provided, cruciate retaining or posterior stabilised implants were used, without reporting the need of using more constraint type of implants.

To reduce bias due to missing data, additional data were obtained from one study [43], after contacting its authors.

### Patient-reported outcome measures

In the six studies [20, 24, 25, 35, 36, 40] reporting PROMs, nine different PROMs were used; the Knee Society Score (KSS), the Western Ontario and McMaster Universities Osteoarthritis index (WOMAC), the Short Form Health Survey-12 (SF-12), the Knee injury and Osteoarthritis Outcome Score (KOOS) (Table 4), the Lower Extremity Activity Score (LEAS), the Visual Analogue Scale (VAS), International Knee Score (IKS), the Oxford Knee Score (OKS), and a 4-level classification of knee pain (Table 5). One study [25] reported statistically significantly lower postoperative KSS and KOOS for all four domains in the PTOA group. Another study [35] reported statistically significantly lower WOMAC knee pain and stiffness scores and higher VAS pain in the PTOA group.

**Table 4:** Patient reported outcome measures in included articles regarding: KSS, WOMAC, SF12, KOOS

(a)KSS							
Study	Study population (PTOA/OA)	Mean interval between fracture and TKA	Mean follow up (PTOA/OA)	Preoperative data		Postoperative data	
				Knee(PTOA/OA)^a^	Functional(PTOA/OA)^a^	Knee(PTOA/OA)^a^	Functional (PTOA/OA)^a^
Lizaur-Utrilla et al (2015)	29/58^b^	6.9 years	6.4/7.1 years	41.5/47.7 n.s.	49.4/54.6 n.s.	86.2/89.1 n.s.	87.9/91.8 n.s.
Lunebourg et al (2015)	33/407^b^	14 years	11 years	31/39 (***p* 0.003**)	44/55 (***p* 0.008**)	77/87 (***p* 0.02**)	81/89 (***p* 0.03**)

(b)WOMAC									
Study	Study population (PTOA/OA)	Mean interval between fracture and TKA	Mean follow up (PTOA/OA)	Preoperative data			Postoperative data		
				WOMAC pain ^(PTOA/OA)a^	WOMAC function ^(PTOA/OA)a^	WOMAC stiffness ^(PTOA/OA)a^	WOMAC pain ^(PTOA/OA)a^	WOMAC function ^(PTOA/OA)a^	WOMAC stiffness ^(PTOA/OA)a^
Lizaur-Utrilla et al (2015)	29/58^b^	6.9 years	6.4/7.1 years	41.8/45.3 n.s.	43.4/47.9 n.s.	N/A	75.4/78.5 n.s.	75.2/79.4 n.s.	N/A
Phruetthiphat et al (2021)	136/716^c^	N/A	8.1/8 years	40.5 /48.7 n.s.	41.2/46.7 n.s.	36.4/40.2 n.s.	70/85.7 (***p* 0.007**)	60.9/71.9 n.s.	50/63.8 (***p* 0.013**)

(c)SF12							
Study	Study population (PTOA/OA)	Mean interval between fracture and TKA	Mean follow up (PTOA/OA)	Preoperative data		Postoperative data	
				Physical ^(PTOA/OA)a^	Mental ^(PTOA/OA)a^	Physical ^(PTOA/OA)a^	Mental ^(PTOA/OA)a^
Khoshbin et al (2019)	75/375^b^	19.3 years	93/88.2 months	35.5/34 n.s.	50.6/52.9 n.s.	46.1/47.5 n.s.	54.8/55.3 n.s.
Lizaur-Utrilla et al (2015)	29/58^b^	6.9 years	6.4/7.1 years	25.8/29.2 n.s.	25.5/28.4 n.s.	41.2/44.6 n.s.	39.9/43.9 n.s.
Scott et al (2015)	31/93^b^	2 years	85 months	31.2/30.7 n.s.	42.3/50.1 (***p* 0.014**)	37.9/42.9 n.s.	50.6/52.3 n.s.

(d)KOOS										
Study	Preoperative data (PTOA^b^/OA)					Postoperative data (PTOA^b^/OA)				
	Pain^a^	Symptoms^a^	ADL^a^	Sports & Recreation^a^	QoL^a^	Pain^a^	Symptoms^a^	ADL^a^	Sports & recreation^a^	QoL^a^
Khoshbin et al (2019)	51.3/49.1 n.s.	48.3/50.4 n.s.	58.5/54.2 n.s.	23.7/21.8 n.s.	23.5/26 n.s.	85.1/88.6 n.s.	80.6/80.6 n.s.	85.6/87.3 n.s.	62.2/61.3 n.s.	71.2/71.1 n.s.
Lunebourg et al (2015)	N/A	N/A	N/A	N/A	N/A	72/86 (***p*<0.001**)	68/85 (***p*<0.001**)	69/85 (***p*<0.001**)	31/70 (***p*<0.001**)	55/78 (***p*<0.001**)

*KOOS* Knee Injury and Osteoarthritis Outcome Score, *KSS* Knee Society Score, *SF-12* The Short Form (12) Health Survey, *WOMAC* Western Ontario and McMaster Universities Osteoarthritis index, *ADL* Activities of Daily Living, *QoL* Quality of Life, *n.s.* Non-significant, *N/A* Not applicable, *bold numbers* p<0.05

^a^Mean value

^b^*PTOA/OA* The group of patients who underwent total knee arthroplasty due to posttraumatic osteoarthritis because of fracture around the knee joint / The group of patients who underwent total knee arthroplasty due to primary osteoarthritis without previous injury around the knee joint

^c^The PTOA group in this case includes patients who underwent total knee arthroplasty due to posttraumatic osteoarthritis not only because of fracture treatment but also due to other injuries such as ligament injuries, high tibial or distal femoral osteotomy, previous open arthrotomy or meniscectomy (the results in this case are presented all together as a hole posttraumatic group and not separately) / The group of patients who underwent total knee arthroplasty due to primary osteoarthritis without previous injury around the knee joint

**Table 5:** Patient reported outcome measures in included articles regarding: LEAS, VAS, IKS, OKS and Level of pain

Study	Study population (PTOA/OA)	Mean interval between fracture and TKA	Mean Follow up (PTOA/OA)								
LEAS											
Khoshbin et al (2019)	75/375^b^	19,3 years	93/88.2 months	Preoperative data (PTOA/OA)^a^				Postoperative data (PTOA/OA)^a^			
				10/9.3 n.s.				11.9/11.6 n.s.			
VAS											
Phruetthiphat et al (2021)	136/716^c^	N/A	8,1/8 years	Preoperative data (PTOA/OA)^a^				Postoperative data (PTOA/OA)^a^			
				8.3/8 n.s.				2.1/1.3 (p 0.019)			
IKS											
Piedade et al (2013)	231/1119^c^	N/A	45 months	Knee score (PTOA/OA) % of improvement				Function score (PTOA/OA) % of improvement			
				91/106 (p 0.01)				42/43 n.s.			
OKS											
Scott et al (2015)	31/93^b^	2 years	85 months	Preop Data (PTOA/OA)^a^		6 months postop (PTOA/OA)^a^		1-year postop data (PTOA/OA)^a^		5 years postop data (PTOA/OA)^a^	
				29.9/18.2 (p 0.001)		33.2/33.5 n.s.		34.4/34.8 n.s.		32.4/35.5 n.s.	
Level of pain											
Piedade et al (2013)	231/1119^c^	N/A	45 months	Preoperative Data (PTOA/OA) %				Postoperative Data (PTOA/OA) %			
				No pain	Slight	Moderate	Severe	No pain	Slight	Moderate	Severe
				1/0.6	19/17 n.s.	60/63 n.s.	20/19 n.s.	50/54 (p 0.023)	42/40 n.s.	8/6 n.s.	0/0

*Preop* preoperative, *Postop* postoperative, *LEAS* Lower Extremity Activity Score, *OKS* Oxford Knee Score, *IKS* International Knee Score, *n.s.* Non-significant

^a^Mean value

^b^*PTOA/OA* The group of patients who underwent total knee arthroplasty due to posttraumatic osteoarthritis because of fracture around the knee joint / The group of patients who underwent total knee arthroplasty due to primary osteoarthritis without previous injury around the knee joint

^c^The PTOA group in this case includes patients who underwent total knee arthroplasty due to posttraumatic osteoarthritis not only because of fracture treatment but also due to other injuries such as ligament injuries, high tibial or distal femoral osteotomy, previous open arthrotomy or meniscectomy (the results in this case are presented all together as a hole posttraumatic group and not separately) / The group of patients who underwent total knee arthroplasty due to primary osteoarthritis without previous injury around the knee joint

### Intraoperative data and complications

Ten studies [6, 10, 11, 13, 19, 21, 35, 36, 40, 49] reported intraoperative data and complications during TKA. In the PTOA group, mean operative time was reported longer consistently in seven studies (Table 6). Two studies [6, 13] reported increased blood loss, one [19] increased rates of blood transfusion and one [40] increased risk for ligament injuries during PTOA TKA.

**Table 6:** Intraoperative data and complications described in included articles

Study	Study population (PTOA/OA)	Mean tourniquet time (min)	Mean operative time (min)	Mean anaesthesia time (min)	Blood transfusion (%)	Mean calculated blood loss (mL)	Ligament injury (%)
Bergen et al (2019)	109/109^c^	90.6/70.7 (**p <0.001**)	147/113.4 (**p <0.001**)	N/A	N/A	225/177 (**p 0.010**)	N/A
Dexel et al (2016)	170/1671^c^	N/A	111.4/87.6 (**p <0.001**)	N/A	N/A	N/A	N/A
El-Galaly et al (2017)	1421/51097^b^	N/A	Range: 94–102/ Range: 72–73 (**p<0.001**)	N/A	N/A	N/A	N/A
Frisch et al (2020)	130/130^c^	N/A	111.2/90 (**p 0.01**)	N/A	1.5/3.1 n.s.	1474.2/1248.7 (**p 0.004**)	N/A
Kester et al (2016)	674/67.675^b^	N/A	121.16/94.5 (**p <0.001**)	186.15/143.34 (**p <0.001**)	20.3/13.5 (**p <0.001**)	N/A	N/A
Kreitz et al (2016)	63/189^c^	N/A	102.1/71.7 (**p< 0.0001**)	N/A	N/A	N/A	N/A
Phruetthiphat et al (2021)	136/716^c^	N/A	125.3/100.1 (**p <0.0001**)	N/A	N/A	175.9/118.5 n.s.	N/A
Piedade et al (2013)	231/1119^c^	N/A	N/A	N/A	N/A	N/A	5.5/4^a^ n.s.
Scott et al (2015)	31/ 93^b^	N/A	N/A	N/A	N/A	N/A	6.45/0 (**p 0.010**)
Wood et al (2022)	1712/1712^b^	N/A	N/A	N/A	3.15/2.62 n.s.	N/A	N/A

*N/A* Not applicable, *n.s. N*on-significant, *bold numbers* p<0.05, *min* Minutes

^a^Includes both ¨ligament injury¨ and ¨fracture¨ subgroups which are described together as one group

^b^*PTOA/OA* The group of patients who underwent total knee arthroplasty due to posttraumatic osteoarthritis because of fracture around the knee joint/The group of patients who underwent total knee arthroplasty due to primary osteoarthritis without previous injury around the knee joint

^c^The PTOA group in this case includes patients who underwent total knee arthroplasty due to posttraumatic osteoarthritis not only because of fracture treatment but also due to other injuries such as ligament injuries, high tibial or distal femoral osteotomy, previous open arthrotomy or meniscectomy (the results in this case are presented all together as a hole posttraumatic group and not separately) / The group of patients who underwent total knee arthroplasty due to primary osteoarthritis without previous injury around the knee joint

### Postoperative complications

All the included studies reported postoperative complications after TKA. These complications were grouped into four categories; infections, soft-tissue complications, revisions and other, based on the terminology used in each study.

#### Infections

Ten studies [6, 17, 19, 21, 25, 27, 29, 40, 43, 49] reported on postoperative infections. The incidence of deep infections was increased (range 1.9–7.9%) in the PTOA group compared with the primary OA group (range 0–3%) with the difference reaching statistical significance in 6 studies (Table 7).

**Table 7:** Postoperative infections^a^

Study	Study population (PTOA/OA)	Deep infection & PJI (PTOA/OA) (%)	Superficial infection (PTOA/OA) (%)	Irrigation & debridement (PTOA/OA) (%)	Sepsis (PTOA/OA) (%)	Wound Complications (PTOA/OA) (%)
Bergen et al (2019)	109/109^c^	7.9/0 (**p 0.005**)	N/A	9/1.1 (**p 0.016**)	1.1/0 (p 0.484)	5.6/0 (**p 0.025**)
Houdek et al (2016)	531/19.641^b^	4.4/1.5 (**p<0.0001**)	2/0.8 (**p<0.0001**)	N/A	N/A	5.3/2 (**p<0.0001**)
Kester et al (2016)	674/67.675^b^	0.148/0.151 n.s.	1.484/0.578 (**p 0.009**)	N/A	N/A	0.89/0.46 n.s.
Kreitz et al (2016)	63/189^c^	3.17/0.529	N/A	0/0.529	1.58/0	N/A
Luneborg et al (2015)	33/407^b^	6.06/0 (**p<0.001**)	0/0.24 (**p< 0.001**)	N/A	N/A	N/A
Manrique et al (2018)	55/110^c^	10.9/4.5 n.s.	N/A	N/A	N/A	1.8/0.9 n.s.
Miozzari et al (2021)	844/3,101^c^	1.9/1.2 (**p<0.001**)	N/A	N/A	N/A	N/A
Scott et al (2015)	31/ 93^b^	3.2/1 n.s.	12.8/1 (**p 0.010**)	N/A	N/A	13/1 (**p 0.014**)
Stake et al (2021)	823/1640^b^	4.74/1.34 (**p<0.001**)	N/A	N/A	N/A	N/A
Wood et al (2022)	1712/1712^b^	5.37/3.03 (**p 0.001**)	N/A	N/A	1.69/1.57 n.s.	2.74/1.34 (**p 0.005**)

N/A Not applicable, *n.s. N*on-significant*, PTOA/OA* The group of patients who underwent total knee arthroplasty due to posttraumatic osteoarthritis because of fracture around the knee joint / The group of patients who underwent total knee arthroplasty due to primary osteoarthritis without previous injury around the knee joint, *PJI* Periprosthetic joint infection, *bold numbers* p value<0.05

^a^In this table we report postoperative infections regardless of whether they led to revision surgery or not

^b^*PTOA/OA* The group of patients who underwent total knee arthroplasty due to posttraumatic osteoarthritis because of fracture around the knee joint / The group of patients who underwent total knee arthroplasty due to primary osteoarthritis without previous injury around the knee joint

^c^The PTOA group in this case includes patients who underwent total knee arthroplasty due to posttraumatic osteoarthritis not only because of fracture treatment but also due to other injuries such as ligament injuries, high tibial or distal femoral osteotomy, previous open arthrotomy or meniscectomy (the results in this case are presented all together as a hole posttraumatic group and not separately) / The group of patients who underwent total knee arthroplasty due to primary osteoarthritis without previous injury around the knee joint

Four studies [17, 19, 25, 40] reported superficial infections. Three of them [17, 19, 40] showed statistically significantly higher rates of superficial infections in the PTOA group and the fourth [25] reported statistically significantly lower rates of superficial infections for PTOA (Table 7).

#### Soft tissue complications

Eight studies [16, 17, 21, 25, 27, 36, 43, 49] evaluated soft-tissue complications postoperatively after TKA, such as “stiffness/arthrofibrosis”, “manipulation under anaesthesia (MUA)”, “patellar instability” and “peroneal nerve palsy”. Two studies [16, 25] reported statistically significantly higher rates of postoperative knee stiffness in the PTOA group (4.4–6.1% vs 0.2–1.1%). MUA was also reported statistically significantly higher for the PTOA group in two studies [16, 17] (Table 8).

**Table 8:** Postoperative complications described in the included studies Subcategory: soft tissue complications

Study	Study population (PTOA/OA)	Stiffness/arthrofibrosis (PTOA/OA) (%)	Manipulation under anaesthesia (PTOA/OA) (%)	Patella Instability (PTOA/OA) %	Peroneal nerve palsy (PTOA/OA) %
Houdek et al (2015)	113/19,641^b^	4.4/1.1 (**p< 0.002**)	9.7/2.4 (**p< 0.0001**)	1.7/0.6 n.s.	N/A
Houdek et al (2016)	531/19.641^b^	N/A	5.6/2.4 (**p< 0.0001**)	N/A	1.6/0.6 (**p< 0.0001**)
Kreitz et al (2016)	63/189^a^	9.52/4.23	9.52/4.23	N/A	N/A
Luneborg et al (2015)	33/407^b^	6.06/0.24 (**p< 0.001**)	N/A	N/A	N/A
Manrique et al (2018)	55/110^a^	7.3/1.8 n.s.	9.1/7.3 n.s.	N/A	N/A
Piedade et al (2013)	231/1119^a^	n.s.	N/A	N/A	N/A
Stake et al (2021)	823/1640^b^	13.24/11.4 n.s.	N/A	N/A	N/A
Wood et al (2022)	1712/1712^b^	4.08/5.43 n.s.	4.9/5.14 n.s.	N/A	N/A

N/A Not applicable, *n.s.* Non-significant, *bold numbers* p value<0.05

^a^ The PTOA group in this case includes patients who underwent total knee arthroplasty due to posttraumatic osteoarthritis not only because of fracture treatment but also due to other injuries such as ligament injuries, high tibial or distal femoral osteotomy, previous open arthrotomy or meniscectomy (the results in this case are presented all together as a hole posttraumatic group and not separately) / The group of patients who underwent total knee arthroplasty due to primary osteoarthritis without previous injury around the knee joint

^*b*^* PTOA/OA* The group of patients who underwent total knee arthroplasty due to posttraumatic osteoarthritis because of fracture around the knee joint / The group of patients who underwent total knee arthroplasty due to primary osteoarthritis without previous injury around the knee joint

#### Revisions

Twelve studies [11, 16, 17, 20, 24, 25, 27, 29, 35, 36, 43, 49] reported on overall TKA revision rates, with ten of these studies [11, 16, 20, 21, 24, 25, 27, 29, 35, 43] also specifying at least one indication for the revision surgery. The studies comprised a variety of estimates for the risk of TKA revision, such as Kaplan–Meier rates, cumulative revision rates, odds ratios, and hazard ratios, as well as a variety of follow ups (Table 9).

**Table 9:** Postoperative complications described in the included studies Subcategory: revisions

Study	TKA revision rate for all causes						
El-Galaly et al (2017)		Total % of revisions (PTOA/OA ^b^)	Entire follow-up		<1y Follow-up	1-5 y Follow-up	>5y Follow up
	**Age** **<50 y**	22/11 (**p<0.001**)	aHR^a^:1.6 (CI: 1.1-2.) (**p=0.01**)		aHR^a^:2.5 (CI:1.4-4.5) (**p 0.002**)	aHR^a^:1.1 (CI: 0.6-2) n.s.	aHR^a^:1.8 (CI: 0.8-4.1) n.s.
	**Age 50-70y**	10/6 (**p<0.001**)	aHR^a^:1.5 (CI:1.2-1.8) (**p<0.001**)		aHR^a^:1.6 (CI:1.1-2.4) (**p 0.02**)	aHR^a^:1.5 (CI:1-2.1) (**p 0.03**)	aHR^a^:1.2 (CI:0.6-2.2) n.s.
	**Age >70y**	7/3 (**p<0.001**)	aHR^a^:1.9 (CI:1.4-2.7) (**p<0.001**)		aHR^a^:1.6 (CI:0.9-3.1) (**p 0.1**)	aHR^a^:2.4 (CI:1.5-3.8) (**p<0.001**)	aHR^a^:1.4 (CI:0.4-4.7) n.s.
Houdek et al (2015)	Survival rates for PTOA / OA ^b^ TKA at 5y:			93% / 97%		HR 0.71 (15y follow-up) n.s.	
	10y:			91% / 93%			
	15y:			86% / 86%			
Houdek et al (2016)	Survival rates for PTOA / OA ^b^ TKA at 5y:			90% / 96%		HR 2.23 (20y follow-up) (**p<0.0001**)	
	10y:			88% / 92%			
	15y:			77% / 85%			
	20y:			67% / 75%			
Khoshbin et al (2019)	*Total % of revisions (PTOA/OA *^*b*^*):* 4% / 3% n.s.			*TKA survival rates (PTOA/OA *^*b*^*):* 96% of 45.2 m / 97% of 69.7 m n.s.			
Lizaur-Utrilla et al (2015)	*Total % of revisions (PTOA/OA *^*b*^*):* 3.44/1.72			*7y TKA survival rates (PTOA/OA *^*b*^*):* 90% (CI: 77%-100%) / 95.2% (CI: 86%-100%) n.s.			
Lunebourg et al (2015)	*Total % of revisions (PTOA/OA *^*b*^*):* 9.09/0 (**p <0.001**)			*10y survival rates for PTOA/OA *^*b*^* TKA:* 94% (CI:89-99)/100% (CI:99-100) (**p 0.002**)			
Manrique et al (2018)	*Overall % aseptic revisions rates (PTOA/OA *^*c*^*):* 7.3/3.6 n.s.			HR ^g^ 1.42 (CI: 0.31-6.41) n.s.			
Miozzari et al (2021)	•*Total % of revisions (PTOA/OA *^*c*^*):* 8.3/4.3 •*HR for all-cause revision:* 2 (CI:1.5-2.8) (10y follow-up) •*aHR* ^d^* for all-cause revision:* 1.6 (CI:1.2-2.2) (10y follow-up)			•5y cumulative failure rates (PTOA /OA ^b^): 8.3% (CI: 3.8-18) / 3.3% (CI: 2.7-4) •5y cumulative failure rates (PTOA /OA ^c^): 6.6% (CI 5.5-8.5) / 3.3% (CI 2.7-4) •10y cumulative failure rates (PTOA/OA ^c^): 8.4% (CI 6.6-11) / 4.5% (CI 3.8-5.4)			
Phruetthiphat et al (2021)	*Total % of revisions (PTOA/OA *^*c*^*):* 0.7/0 n.s.						
Piedade et al (2013)	*Total % of revisions (PTOA/OA *^*c*^*):* 37.5/41 n.s.			*120m Kaplan-Meier survival curve rates for PTOA / OA *^*c*^* TKA* 90.9% (CI: 100-77.6%) / 98.1% (CI: 99.1-97.1%) n.s.			
Stake et al (2021)	*Total % of revisions (PTOA/OA *^*b*^*):* 5.47/2.74 (**p 0.001**)						
Wood et al (2022)	*Total % of revisions (PTOA/OA *^*b*^*):* •1y follow-up: 2.1/1.75 n.s. •2y follow-up: 3.1/2.27 n.s.			*Odds ratio (OR):* •1y follow-up: OR=1.204 (CI: 0.738-1.964) n.s. •2y follow-up: OR=1.496 (CI: 0.983-2.277) n.s.			

Study	Study population (PTOA/OA)	TKA revision rates due to specific causes (PTOA/OA%)				
		Aseptic Loosening	Infection	Instability	Pain	Periprosthetic fracture
El-Galaly et al (2017)	1421/51097^b^	3.2/1	3.2/1.4	3.5/1.1	1.4/0.75	N/A
Houdek et al (2015)	113/19,641^b^	0.9^e^/0.3^e^ n.s.	N/A	N/A	N/A	N/A
Khoshbin et al (2019)	75/375^b^	0/0.8 n.s.	2.66/0.533 n.s.	1.33/0.8 n.s.	N/A	N/A
Kreitz et al (2016)	63/189^c^	N/A	3.17/0^f^	N/A	N/A	N/A
Lizaur-Utrilla et al (2015)	29/58^b^	3.44/1.72 n.s.	N/A	N/A	N/A	N/A
Luneborg et al (2015)	33/407^b^	3.03/0 (**p<0.001**)	6.6/0 (**p<0.001**)	N/A	N/A	N/A
Manrique et al (2018)	55/110^c^	5.5/0 (**p 0.01**)	N/A	N/A	N/A	N/A
Miozzari et al (2021)	844/3,101^c^	2.1/0.9	1.9/1.2	0.4/0.2	0.9/0.5	0.6/0.4
Phruetthiphat et al (2021)	136/716^c^	0.7^g^/0 n.s.	N/A	N/A	N/A	N/A
Stake et al (2021)	823/1640^b^	1.82/0.91 n.s.	N/A	N/A	N/A	N/A

*N/A* Not applicable, *n.s.* Non-significant, *y* Years, *m* Months, *PTOA/OA* The group of patients who underwent total knee arthroplasty due to posttraumatic osteoarthritis because of fracture around the knee joint/The group of patients who underwent total knee arthroplasty due to primary osteoarthritis without previous injury around the knee joint, *aHR* Adjusted hazard ratios, *HZ* Hazard ratios, *OR* Odds ratio, *CI* 95% confidence interval, bold numbers p value<0.05

^a^Hazard ratio for the PTOA group relative to the OA group, adjusted for sex, weight, Charnley class, perioperative complications, and the need for additional component supplementation

^b^PTOA/OA The group of patients who underwent total knee arthroplasty due to posttraumatic osteoarthritis because of fracture around the knee joint / The group of patients who underwent total knee arthroplasty due to primary osteoarthritis without previous injury around the knee joint

^c^The PTOA group in this case includes patients who underwent total knee arthroplasty due to posttraumatic osteoarthritis not only because of fracture treatment but also due to other injuries such as ligament injuries, high tibial or distal femoral osteotomy, previous open arthrotomy or meniscectomy (the results in this case are presented all together as a hole posttraumatic group and not separately) / The group of patients who underwent total knee arthroplasty due to primary osteoarthritis without previous injury around the knee joint

^d^Adjusted for age, sex, ASA score and year of surgery

^e^Referring only to patella component loosening

^f^This study reported only 2-stage TKA revisions because of infection as result in the revision group, without presenting the total number of TKA revision rate for other causes

^g^Only referring to revision cases due to aseptic loosening, information regarding the specific time interval when calculating Hazard ratio is not provided in the original article

The estimates of revision risk were consistently increased in the PTOA group compared with primary OA at almost all follow ups and in all twelve studies. The increased revision risk had a varying magnitude (e.g. hazard rations for PTOA TKA revision varied from 1.1 to 2.5) and reached statistical significance in five studies [11, 17, 25, 29, 43] (Table 9). More specifically, El Galaly et al. (2017) [11] reported an increased risk of early and medium-term revision after TKA due to PTOA compared with the primary OA. This tendency continued even in the longer-term follow-up, albeit without reaching statistical significance. Houdek et al. (2016) [17] also reported a significantly increased risk of revision TKA in the PTOA group, with approximately one in four patients requiring revision by 15 years. The fracture location did not affect the risk of TKA revision.

Regarding specific reasons for TKA revision, aseptic TKA loosening, postoperative infections and TKA instability were overrepresented in the PTOA group [25, 27] (Table 9).

#### Other complications

Ten studies [6, 13, 16, 17, 19, 21, 35, 36, 43, 49] reported on other complications such as “deep venous thromboembolism/pulmonary embolism (DVT/PE)”, “readmission rate 90 days postoperatively”, “mortality”, “periprosthetic fracture” and “another additional procedure”. None of them reported any statistically significant difference in the risk of DVT/PE or in mortality rates. Increased readmission rates were reported in one study [6] for the PTOA group (15% vs 4%, *p* 0.02). Increased risk for periprosthetic fractures after PTOA TKA were reported in two studies (4.5% vs 1.3%, *p* < 0.001 [17] and 1.6% vs 0.6%, *p* 0.01 [43]). Finally, two studies [17, 19] reported an increased risk for the need of additional surgical procedure.

## Discussion

The main finding of the present systematic review was the higher rates of complications after TKA in patients with PTOA due to previous fracture treatment compared with patients who received TKA due to primary OA. Additionally, a tendency towards a poorer preoperative and postoperative PROMs was shown in the PTOA group in comparison with the primary OA group, in line with our hypothesis, however, this tendency is difficult to generalise due to the inconsistent presence of statistical significance in the literature.

The findings in this review are similar to the results of previous studies that reported poorer preoperative scores in the PTOA group, emphasising the importance of considering the preoperative status in addition to the intrinsic success of the TKA intervention postoperatively. In the same manner, another study used this explanation to support its oppositional findings, that patients with primary OA reported poorer preoperative Oxford Knee Scores compared with the PTOA group, but the improvements within both cohorts were found to be the same. However, the small sample size of this study needs be taken into consideration. On the other hand, a larger study with no differences in preoperative PROMs between the cohorts reported greater postoperative pain measured with a VAS, as well as statistically significantly lower WOMAC pain and stiffness items postoperatively in patients with PTOA after fracture compared with primary OA patients. This observation may support the hypothesis that patients with fracture treatment prior to TKA sustain postoperative complications that are associated with pain, more frequently than OA patients.

Intraoperative complications were not comprehensively and consistently examined in the included studies. The most interesting finding was that the mean operative time was consistently longer in the PTOA group in all studies [6, 10, 13, 19, 21, 35] that reported this outcome. One possible explanation could be the need to remove osteosynthesis material, as well as the management of changes in anatomy and previous scars from previous surgical procedures [13]. As a result, soft-tissue balancing may be challenging and, apart from increased surgical skills, it may require the use of more sophisticated TKA systems than those commonly used in primary OA. An extended surgical procedure could put the patients at risk due to prolonged anaesthesia time and increase the risk of postoperative infection [8, 30]. In accordance with the results of the present review, a recent systematic review and meta-analysis of the topic has demonstrated a strong association between prolonged operative time and complications. In fact, the risk of developing complications, such as infections, renal failure and cardiovascular complications, increased exponentially with increased operative time [9]. This finding underlines the importance of detailed preoperative planning, in order to prevent unexpected problems that could prolong the duration of surgery. In addition, further research is warranted, focussing on the development of surgical techniques, such as robot-assisted TKA and patient-specific instrumentation that aim to increase the accuracy of implant placement and reduce the operative time in complex TKA.

The present review demonstrates higher postoperative complication rates in patients receiving TKA after PTOA with prior fracture treatment compared with primary OA in all the included studies. These results are comparable with those observed in previous studies [23, 38]. One interesting finding was the overall increase in infection rates after TKA in the PTOA group. This could be explained by the prolonged operative time, as previously discussed. The presence of contaminated osteosynthesis material and bone or soft-tissue necrosis caused by the previous trauma and/or surgery may also be associated with the increased risk of infection after TKA. [39]. Further, the bioenvironmental complexities in a knee with previous surgery may also serve as a reasonable explanation for the higher rate of implant loosening, as well as soft-tissue complications in the PTOA group, such as postoperative stiffness and patellar instability [11, 16, 25, 27, 29, 49]. Finally, all the studies reporting TKA revisions demonstrated lower implant survival rates in the PTOA group compared with the OA group, but not always reaching statistical significance. Aseptic loosening and infection were the most common causes of revision in both groups, with estimates favouring the OA group. This disparity could be explained by the intrinsic environmental changes affecting the knee globally due to previous trauma or surgery that could imply poorer bone quality, retained osteosynthesis material, a compromised soft-tissue envelope, adhesions, and deformity [40, 43]. Consequently, less favourable settings for a knee replacement are created, which makes the procedure more difficult in terms of restoring knee alignment, implant positioning and soft tissue balancing. Apart from the abovementioned issues with PTOA TKA, another aspect to be taken into consideration is that PTOA generally occurs more frequently in younger patients [25, 29]. They often have a higher activity level, which increases the cumulative stress on the implant and surrounding tissues. The combination of increased implant stress and the aforementioned factors including knee alignment and implant positioning matters as well as postoperative infections could lead to early TKA failure. As a result, the risk of TKA revision is reportedly higher in patients with PTOA [14, 15, 28].

To summarise, the findings in the present review could suggest that the increase in postoperative complication rates after PTOA TKA may be a result of the inherent technical challenges of the TKA procedure that includes preoperative status and patient-related factors that place greater demands on the surgeon’s capability to perform the procedure. However, information on institutional differences in volume and surgeon experience is limited in the literature. This could be a relevant covariable to adjust for in the future studies to elucidate the actual impact of PTOA after fracture treatment for TKA.

### Limitations

There are limitations to the present study. First, the number of studies fulfilling the inclusion criteria was quite low, which makes the possibility of not obtaining fully representative results more likely. Even though an extensive electronic search was performed in four different databases (PubMed, Cochrane Library, Scopus and EMBASE), it is possible that some studies were not discovered. Furthermore, the fact that only studies written in English were included could create a language and publication bias. The retrospective character of most of the included studies [6, 10, 13, 16, 17, 20, 21, 25, 27, 35, 36, 43] could introduce bias inherent to the nature of the study design, since factors that could potentially confound the effect of PTOA on the outcome of TKA remain unknown and could not be taken into account. In order to comply with this retrospective methodology, some studies [6, 20, 21, 43] used the International Classification of Diseases (ICD) or the Current Procedural Terminology (CPT) codes for data capture, a method that is highly dependent on the consistency and reliability of these codes and patients being coded correctly. Additionally, nine of the eighteen included studies [6, 13, 21, 24, 25, 27, 35, 36, 40] had a relatively small sample size, which may have underpowered these studies to detect significant differences between the two groups. Some of the studies [6, 13, 19] reported a relatively short follow-up time (mean follow-up time: 3 months), which is considered inadequate to assess long-term postoperative complications.

Furthermore, the chronological period of patient recruitment in some of the included studies [16, 17, 25, 36, 40] is more than 15 years, a fact that could represent a confounding factor to this review analysis due to outdated types of implants, surgical techniques and/or alignment strategies.

Finally, there was heterogeneity in PROMs, reported complications and terminology used for each complication, as well as differences in the follow-up time across the included studies. This highlights the challenges of pooling results when conducting systematic reviews and meta-analyses, possibly warranting the need for consensus in the scientific community regarding the choice of PROM instruments for measuring the outcome after surgical TKA.

## Conclusions

PROM analysis suggests that both patient groups benefit from a TKA in terms of functional outcome and pain relief, however, patient-reported outcomes could be inferior for PTOA patients. There is consistent evidence for increased complication rates following PTOA TKA. Patients undergoing TKA due to PTOA after fracture treatment should be informed about the risk for inferior results and refrain from comparing their knee function to patients with TKA after OA. Surgeons should be aware of the challenges that PTOA TKA poses.

## Supplementary Information

Below is the link to the electronic supplementary material.

Supplementary file 1 (DOCX 21 KB)

## Data Availability

Data obtained during the literature review by the independent authors are available upon request.

## Abbreviations

ACL: Anterior cruciate ligament
ADL: Activities of daily living
aHR: Adjusted hazard ratio
CASP: Critical Appraisal Skills Programme
CI: Confidence interval
CPT: Current procedural terminology
DOI: Digital object identifier
DSI: Deep site infection
DVT: Deep vein thrombosis
HR: Hazard ratio
IC: Intraoperative complications
ICD: International Classification of Diseases
IKS: International Knee Score
ISSN: International standard serial number
JR: Joint replacement
KOOS: Knee Injury and Osteoarthritis Outcome Score
KSS: The Knee Society Score
LEAS: Lower Extremity Activity Score
LOS: Length of stay
LoE: Level of evidence
m: Months
min: Minutes
MUA: Manipulation under anaesthesia
NA: Not applicable
NS: Not significant
OA: Osteoarthritis
OKS: Oxford Knee Score
OR: Odds ratio
ORIF: Open reduction and internal fixation
P: Prospective
PC: Postoperative complications
PE: Pulmonary embolism
PECO: Patients—Exposure—Comparator—Outcome
PJI: Periprosthetic joint infection
PNP: Peroneal nerve palsy
PP: Periprosthetic
PRISMA: Preferred Reporting Items for Systematic Reviews and Meta-Analyses
PROMs: Patient reported outcome measures
PTOA: Posttraumatic osteoarthritis
QoL: Quality of Life
R: Retrospective
RCT: Randomised controlled trials
ROM: Range of motion
RR: Revision rate
SF-12: Short Form Health Survey-12
TKA: Total knee arthroplasty
VAS: Visual Analogue Scale
WC: Wound complications
WOMAC: The Western Ontario and McMaster Universities Osteoarthritis index
Y: Years
