# Supplementary material for: Increased complications rates and inferior patient reported outcomes following total knee arthroplasty due to post-traumatic osteoarthritis with previous fracture treatment: a systematic review
Source: Knee Surg Sports Traumatol Arthrosc. 2023 Apr 25;31(10):4124–41. doi: 10.1007/s00167-023-07407-x (PMC10471648; doi:10.1007/s00167-023-07407-x)
Supplement: Supplementary file 1 — (DOCX 21 KB) [file 167_2023_7407_MOESM1_ESM.docx]

**Appendix A:** Search syntax for PubMed, Cochrane Library, Scopus and EMBASE

| **#** | **PubMed** search done 11-01-2021 and updated search done 23-11-2022  **Searches:** | **Results** | |
| --- | --- | --- | --- |
| 1 | Proximal tibial fracture*[tw] OR Tibial condyle fracture*[tw] OR Tibial plateau fracture*[tw] OR Distal femoral fracture*[tw] | 2 217 |  |
| 2 | tibial fractures[mh] OR femoral fractures[mh] | 55 974 |  |
| 3 | Osteosynthesis[tw] OR((internal[tw] OR external[tw]OR intramedullary nail[tw] OR plate[tw]) AND fixat*[tw]) OR Fracture Fixation, Internal[mh] | 86 245 |  |
| 4 | #1 OR #2 OR #3 | 121 774 |  |
| 5 | (Total Knee Arthroplasty[tiab] OR TKA [tiab] OR Arthroplasty, Replacement, Knee*[mh]) | 35 185 |  |
| 6 | #4 AND #5 | 1 263 |  |
| 7 | Limit year 2000- | **1 130** |  |

| **#** | **Scopus** search done 11-01-2021 and updated search done 23-11-2022  **Searches:** | **Results** |
| --- | --- | --- |
| 1 | TITLE-ABS-KEY (“Proximal tibial fracture*” OR “Tibial condyle fracture*” OR “Tibial plateau fracture*” OR “Distal femoral fracture*” OR “tibial fracture*” OR “Segond Fracture*” OR “Toddler's Fracture*” OR “Toddler Fracture*” OR “Toddlers Fracture*” OR “Tillaux Fracture*” OR “femoral fracture*”) | 35 532 |
| 2 | TITLE-ABS-KEY (Osteosynthesis OR Osteosyntheses OR ((internal OR external OR “intramedullary nail” OR plate) AND fixat*) OR “internal Fracture Fixation”) | 109 563 |
| 3 | #1 OR #2 | 130 189 |
| 4 | TITLE-ABS-KEY (“Total Knee Arthroplasty” OR TKA OR “Arthroplasty, Replacement, Knee*” OR “Knee Replacement Arthroplasty” OR “Total Knee Replacement” OR “Knee Arthroplasty” OR “knee replacement”) | 53 30 |
| 5 | #3 AND #4 | 2 134 |
| 6 | Limit year 2000- | **1 844** |

| **#** | **Cochrane** search done 11-01-2021 and updated search done 23-11-2022  **Searches:** | **Results** |
| --- | --- | --- |
| 1 | “Proximal tibial fracture*” OR “Tibial condyle fracture*” OR “Tibial plateau fracture*” OR “Distal femoral fracture*” OR “tibial fracture*” OR “Segond Fracture*” OR “Toddler's Fracture*” OR “Toddler Fracture*” OR “Toddlers Fracture*” OR “Tillaux Fracture*” OR “femoral fracture*” OR Osteosynthesis OR Osteosyntheses OR ((internal OR external OR “intramedullary nail” OR plate) AND fixat*) OR “internal Fracture Fixation” | 5 948 |
| 2 | “Total Knee Arthroplasty” OR TKA OR “Arthroplasty, Replacement, Knee*” OR “Knee Replacement Arthroplasty” OR “Total Knee Replacement” OR “Knee Arthroplasty” OR “knee replacement” | 8 943 |
| 3 | #1 AND #2 | 86 |
| 4 | Limit year 2000- | **83** |

| **#** | **Embase** search done 14-01-2021 and updated search done 23-11-2022  **Searches:** | **Results** |
| --- | --- | --- |
| 1 | (proximal tibial fracture* or tibial condyle fracture* or tibial plateau fracture* or distal femoral fracture* or tibial fracture* or segond fracture* or toddler's fracture* or toddler fracture* or toddlers fracture* or tillaux fracture* or femoral fracture* or osteosynthesis or osteosyntheses).tw. | 26 271 |
| 2 | exp tibia fracture/ | 17 772 |
| 3 | exp femur fracture/ | 31 964 |
| 4 | exp osteosynthesis/ | 43 446 |
| 5 | (internal or external or intramedullary nail or plate).tw. | 1 021 600 |
| 6 | "fixat*".tw. | 188 460 |
| 7 | 5 and 6 | 51 311 |
| 8 | 1 or 2 or 3 or 4 or 7 | 113 074 |
| 9 | (total knee arthroplasty or TKA or knee replacement arthroplasty or total knee replacement or knee arthroplasty or knee replacement or Knee replacement).ab,kf,ti. | 44 064 |
| 10 | exp knee replacement/ | 19 032 |
| 11 | 9 or 10 | 47 015 |
| 12 | 8 and 11 | 1948 |
| 13 | limit 12 to yr="2000-Current" | **1738** |

exp tibia fracture/(meshterm: Tibial Fractures[mh])
exp femur fracture/(meshterm: Femoral Fractures[mh])
exp osteosynthesis/(meshterm: Fracture Fixation, Internal[mh])
exp knee replacement/(meshterm: Arthroplasty, Replacement, Knee[mh]
